# Inhibition of HMGB1 Suppresses Hepatocellular Carcinoma Progression *via* HIPK2-Mediated Autophagic Degradation of ZEB1

**DOI:** 10.3389/fonc.2021.599124

**Published:** 2021-03-04

**Authors:** Wei Zhu, Jun Li, Yuheng Zhang, Zhengyi Zhu, Hanyi Liu, Yunzhen Lin, Anyin Hu, Jingchao Zhou, Haozhen Ren, Xiaolei Shi

**Affiliations:** ^1^ Department of Anesthesiology, Affiliated Drum Tower Hospital of Nanjing University Medical School, Nanjing, China; ^2^ Department of General Surgery, The First Affiliated Hospital of Anhui Medical University, Hefei, China; ^3^ Department of Hepatobiliary Surgery, Affiliated Drum Tower Hospital of Nanjing University Medical School, Nanjing, China

**Keywords:** hepatocellular carcinoma (HCC), high-mobility group box 1 (HMGB1), epithelial-mesenchymal transition (EMT), autophagy, glucose metabolism

## Abstract

Autophagy is a conserved catabolic process maintaining cellular homeostasis and reportedly plays a critical role in tumor progression. Accumulating data show that autophagic activity is inhibited in hepatocellular carcinoma. However, the underlying molecular basis of impaired autophagy in HCC remains unclear. In this study, we revealed that autophagic activity was suppressed by HMGB1 in a HIPK2-dependent way. Targeting HMGB1 could inhibit the degradation of HIPK2, as a result of which, autophagic degradation of ZEB1 was enhanced by reprogramming glucose metabolism/AMPK/mTOR axis. Moreover, we demonstrated that selectively degradation of ZEB1 was responsible for HCC growth inhibition in HMGB1 deficient cells. Lastly, we found the combination therapy of HMGB1 inhibitor and rapamycin achieved a better anti-HCC effect. These results demonstrate that impaired autophagy is controlled by HMGB1 and targeting HMGB1 could suppress HCC progression *via* HIPK2-mediated autophagic degradation of ZEB1.

## Introduction

Hepatocellular carcinoma (HCC) is the fifth most common malignancy but the third leading cause of cancer-related deaths worldwide (1). Most HCC cases are closely associated with chronic liver inflammation caused by infections with hepatitis B or C virus, alcoholic liver disease, and nonalcoholic steatohepatitis. The 5-year survival rate of patients that accepted a curative surgery was 18% (2, 3). The poor prognosis of patients with HCC is majorly because of tumor progression such as intrahepatic and distant metastasis (4). Therefore, determining the underlying mechanisms involved in HCC is extremely important.

Autophagy is a conserved catabolic pathway for removing and recycling macromolecules and damaged organelles by fusing with lysosomes (5–7). Autophagy related degradation could be divided into two types: non-selective and selective degradation, both of which were reported to be important for cellular homeostasis and various diseases (5, 8). Impaired autophagic activity has been demonstrated in aging, neurodegenerative diseases, metabolic diseases, and tumorigenesis (9–11). Accumulating data indicate that autophagy is suppressed and plays a crucial role in HCC progression (12–15). Autophagy impairment in HBV-positive HCC was shown to be correlated with tumor progression (16, 17). Moreover, several drugs such as sorafenib, Metformin, rapamycin and HDAC inhibitors were demonstrated to inhibit HCC by triggering autophagy (18–22). Therefore, re-activating autophagy could be an efficient treatment for HCC.

High-mobility group box 1 (HMGB1), a conserved nuclear chromatin-binding protein that acts as a DNA chaperone, is critical for maintaining genome stability and transcription (23–26). HMGB1 has been identified as a component of damage-associated molecular patterns, which have complicated roles in many diseases, including cancers (27, 28). Mounting evidence revealed that HMGB1 was an important tumor-promoting effector in HCC and targeting HMGB1 could significantly restrain HCC progression (29, 30). Moreover, HMGB1 was shown to modulate autophagy by binding to beclin-1 and controlling p53 nuclear translocation (31–33). However, little is known about the relationship between HMGB1 and impaired autophagy in HCC.

The present study showed that HMGB1 expression was inversely correlated with levels of autophagy in HCC specimens. We demonstrated that HMGB1 was essential for HIPK2 degradation and targeting HMGB1 significantly induced autophagy in a HIPK2-dependent manner. Reprogramming of glucose metabolism in HMGB1 deficient HCC cells was caused by HIPK2-mediated p53 nuclear translocation, which further led to autophagy upregulation by activating AMPK/mTOR signaling pathway. Moreover, we revealed that autophagic degradation of ZEB1 was responsible for HMGB1 deficiency-mediated HCC growth inhibition. Lastly, rapamycin, an autophagy inducing drug, enhanced the anti-HCC effects of HMGB1 inhibitor glycyrrhizin by facilitating the degradation of ZEB1. Taken together, we demonstrate the critical role of HMGB1 in impaired autophagy and targeting HMGB1 could suppress HCC progression through reactivating autophagic degradation of ZEB1 in a HIPK2-dependent way.

## Materials and Methods

### Patients and Specimens

Tumor samples were achieved from 76 patients who had undergone curative resection between 2014 and 2016 and were pathologically confirmed HCC at Medical School of Nanjing University Affiliated Drum Tower Hospital. Written informed consent was obtained from the patients prior to the commencement of the experiments and the study protocol was approved by the Review Board of Medical School of Nanjing University Affiliated Drum Tower Hospital. The clinical signatures of all patients are summarized in **Supplementary Table 1**.

### Animals and Chemical Reagents

Male BALB/c nu/nu mice (6–8 weeks old, Shanghai Institute of Material Medicine, Chinese Academy of Science) were housed in specific pathogen-free conditions. All animals received humane care according to the criteria outlined in the “Guide for the Care and Use of Laboratory Animals” prepared by the National Academy of Sciences and published by the National Institutes of Health (NIH publication 86-23 revised 1985). 3-MA (No. S2767), rapamycin (No. S1039), and glycyrrhizin (No. S2302) were purchased from Selleck Chemicals (Houston, TX, USA). The Cell Counting Kit-8 (CCK-8) kit was purchased from Dojindo Laboratories (Kumamoto, Japan).

### Cell Culture

The human HCC cell line HCCLM3 and Bel7402 was achieved from the Cell Bank of the Chinese Academy of Sciences (Shanghai, China). Both cell lines were cultured as previously described (19). HCCLM3 and Bel7402 testing was routinely performed by STR profile and was negative in our previous study (19).

### GO Assays

Glucose concentration in cultural medium was tested by GO assays (Sigma-Aldrich, USA) in accordance with protocol.

### Lactate Assays

Lactate concentration in cultural medium was tested by lactate assays (Biovision, USA) in accordance with protocol.

### HCC Orthotopic Models *In Vivo*

HCCLM3 cells were subcutaneously injected into nude mice. After 15 days, the tumor mass was removed and minced into small pieces (2 × 2 × 2 mm^3^), and transplanted into the livers of normal nude mice. Those nude mice were divided into three groups including control, Glycyrrhizin and Glycyrrhizin+Rapamycin. Four weeks after implantation, the livers bearing tumors were obtained and shown. n = 5. The liver tumor volume was calculated by V=(length x width^2^)/2.

### Immunoblot Analysis

Immunoblot analysis was used to analyze protein expression as described previously (19). Briefly, total protein was extracted by lysing cells in RIPA buffer containing protease inhibitor cocktail. Protein samples boiled with 1x loading buffer were separated by sodium dodecyl sulfate polyacrylamide gel electrophoresis (SDS-PAGE) and transferred onto polyvinylidene fluoride (PVDF) membranes. After blocking with 5% BSA in TBS-T, membranes were incubated with the primary antibody at 4°C overnight. Goat-anti-rabbit or mouse IgG conjugated to horseradish peroxidase (HRP) was used as the secondary antibody. Signal was detected with enhanced chemiluminescence (Millipore) according to the manufacturer’s instructions. Results were imaged by Tanon system and analyzed by ImagePro Plus software.

### Q-PCR

Total RNA of tumor tissues from HCC patients was isolated using Trizol reagent (Life Technology, USA). 2 μg of total RNA were reverse-transcribed (Takara, Kyoto, Japan). The specific primers used to amplify relevant genes are shown in **Supplementary Table 2**. The PCR was carried out in triplicate using SYBR Green real-time PCR master mix (Takara) in an ABI. All results are normalized to 18S rRNA expression.

### RNAi and Gene Transfection

For stably knockdown of HMGB1 with lenti-virus shRNA, 2 × 105 cells were planted onto 6-well plates. After 24 h, the liquid containing shRNA was added to the cultural medium according to protocol. To select stable transfectants, cells were cultured in complete DMEM with 10 μg/ml puromycin (Sigma-Aldrich, USA) for some weeks. HIPK2 siRNA, AMPK siRNA, Siah2 siRNA, p53 siRNA, HMGB1 siRNA, and control siRNA (Riobio, China) were transfected into cells using Lipofectamine 3000 (Invitrogen, USA) according to the manufacturer’s instructions. At the end of the siRNA treatment (48-72 h), the cells were collected for immunoblot analysis and Q-PCR. Expression plasmids for human HMGB1-cDNA (pEnter) and ZEB1-cDNA (pEnter) were purchased from Vigene Biosciences, lnc. For the overexpressing or rescue experiments, HMGB1-cDNA, and ZEB1-cDNA were transfected into a standard or stable shRNA cell line by Lipofectamine 3000 according to the manufacturer’s instructions.

### Immunofluorescence

Immunofluorescence analysis was processed according to protocols. Cells or HCC frozen tissues were fixed by 4% paraformaldehyde 24 h later. Fixed cells or tissues were stained with autophagy related proteins (Cell Signaling Technology, USA), EMT related proteins (Cell Signaling Technology, USA) and p53 (Bioworld, China), followed by FITC–conjugated anti–mouse IgG and Cy3–conjugated anti–rabbit IgG (abcam, USA). Representative images were detected by fluorescent microscopy (Leica, Germany) and data were processed *via* ImagePro Plus software.

### Immunohistochemistry

Immunohistochemistry of HCC samples were conducted as previously described. Briefly, after incubation with HMGB1 (abcam, USA), EMT related markers (Cell Signaling Technology, USA), autophagy related markers (Cell Signaling Technology, USA), p-AMPK (Cell Signaling Technology, USA), mTOR (Cell Signaling Technology, USA), or HIPK2 (abcam, USA), the sections were stained in an Envision System (DakoCytomation, USA). IHC results were scored according to +,<25%; ++, <50%; +++, <75%; ++++, >75% by two experienced pathologists. Data are shown as means ± SEM.

### Invasion Assays

The invasive ability of HCC cells was measured *via* 24-well transwell chambers separated by polycarbonate membranes with 8-µm pores and precoated with Matrigel (Corning, USA). The lower chamber was filled with complete DMEM as a chemoattractant. Cells in serum-free medium were seeded at 5 × 104 in the upper chamber and incubated at 37°C in a humidified incubator containing 5% CO2. Cells that migrated to the underside of the membrane were fixed and stained with Giemsa (Sigma-Aldrich, USA), detected, and calculated with a microscope (Leica, Germany). All experiments were carried out in triplicate.

### Co-Immunoprecipitation

Bel7402 and HCCLM3 cells either transfected with HMGB1 shRNA or negative control was harvested and lysed in ice-cold IP buffer (Keygene, China) for 30 min. Total protein extracts were centrifuged at 12,000 g for 10 min at 4°C. 500 µl of the total lysate was incubated at 4°C with 2 μg of corresponding antibodies or IgG as control and 50 μl protein A/G beads (Santa Cruz, USA) to immunoprecipitate zeb1, p62, lc3II, and slug. The interacted complexes were then washed three times with lysis buffer. After centrifuged at 12,000 g for 10 min at 4°C, pellets were suspended in 100 µl lysis buffer and boiled with 1x SDS loading buffer and then processed by immunoblot analysis.

### Transmission Electron Microscopy

Cells seeded onto 6-well plate were fixed with fixative buffer containing 2% paraformaldehyde and 2.5% glutaraldehyde in 0.1 M PBS. After embedded, samples were cut into 0.12-μm sections and stained with 0.2% lead citrate and 1% uranyl acetate. The images were detected by a JEOL TEM-2000 EX II (JEOL, Tokyo, Japan).

### Statistical Analysis

Fisher’s exact tests and χ2 tests were used to determine clinicopathological correlations. The association between HMGB1, EMT markers, and ZEB1 in HCC tissues was evaluated by Spearman’s correlation. GraphPad Prism 6 was used for all statistical analyses. P < 0.05 was considered statistically significant.

## Results

### HMGB1 Promotes Disease Progression of Human HCC and Is Inversely Associated With Levels of Autophagic Activity

To investigate the expression and role of HMGB1 in HCC, 76 HCC surgical specimens were used to determine HMGB1 expression levels by immunohistochemistry. Our statistical evaluation showed that HMGB1 expression was varied and significantly associated with HCC progression, including TNM stage, recurrence, and PVTT (**Figures S1A, B**). These findings confirmed that HMGB1 is a tumor-promoting effector in HCC.

**Figure 1:**
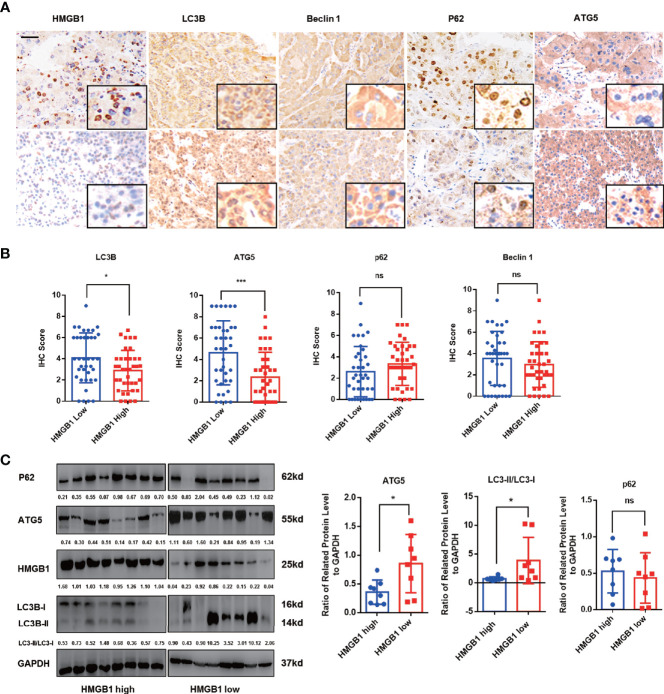
HMGB1 negatively correlates with autophagy markers in HCC. **(A)** Representative images of immunohistochemical (IHC) staining of HMGB1 and autophagy markers in HCC tissues, scale is 100 μm. **(B)** Analysis of IHC scores of autophagy markers according to HMGB1 expression in HCC specimens. HMGB1 low, IHC score 0–1; HMGB1 high, IHC score 2–3. **(C)** Immunoblot analysis of levels of HMGB1 and autophagy markers in 16 HCC patients. Related protein levels were quantified and analyzed based on HMGB1 expression. Data are means ± SEM from three independent experiments, NS means no significance. * means p<0.05, *** means p<0.001 by unpaired student T test.

Autophagy has been reported to be suppressed in HCC and dysfunctional autophagy accounts for HCC progression (11, 12, 14, 17). We observed that HCC patients with higher autophagic activity obtained better prognosis and found expression of autophagy marker LC3B was significantly inhibited in HCC tissues (**Figures S2A, B**). To explore the potential relationship of HMGB1 and levels of autophagy, autophagy related markers such as LC3B, ATG5, P62 and Beclin-1 were stained in HCC samples. Interestingly, IHC results revealed that HMGB1 expression was negatively correlated with autophagy markers LC3B and ATG5 (**Figures 1A, B**). To further demonstrate the relationship between HMGB1 and autophagy, immunoblot analysis was carried out in 16 HCC samples. The ratio of LC3-II/LC3-I and ATG5 were both used to measure the level of autophagy. Though there were no statistically significances between HMGB1 and p62, it seemed that relationship between HMGB1 and autophagy level measured by LC3-II/LC3-I and ATG5 was inversely relevant (**Figure 1C**).

### Targeting HMGB1 Promotes Autophagic Activity Through Stimulating AMPK/mTOR Signaling Pathway

**Figure 2:**
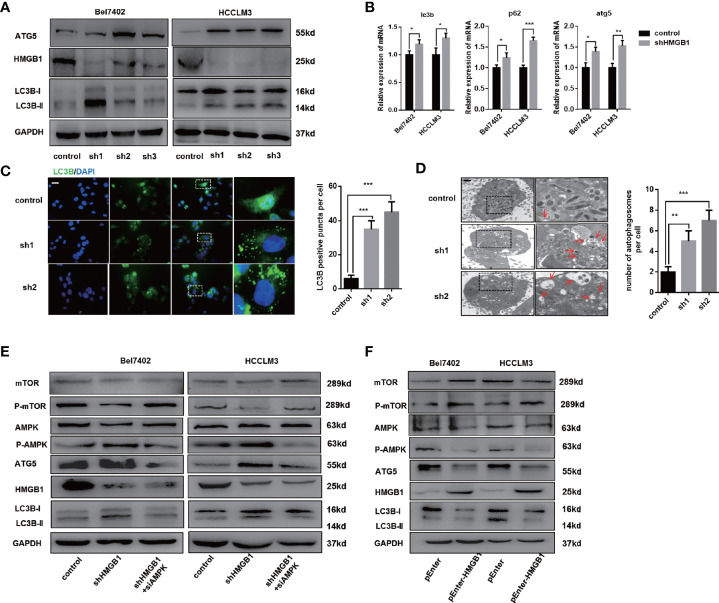
HMGB1 regulates autophagy levels through AMPK/mTOR signaling. **(A)** Protein levels of HMGB1 and autophagy makers were determined in two HCC cell lines with a non-targeting control lentivirus-shRNA (control) or three lentivirus-shRNA targeting HMGB1 mRNA (sh1, sh2, and sh3). Related protein levels were quantified and analyzed. **(B)** Q-PCR experiments were performed to detect mRNA expressions of autophagy markers in both Bel7402 and HCCLM3 cell lines with or without HMGB1 lentivirus-shHMGB1 transfection (shHMGB1). **(C)** Representative images of immunofluorescence (IF) staining LC3B in Bel7402 control, sh1, and sh2 cells. Scale is 100 μm. Numbers of LC3B puncta in three groups were quantified and analyzed. **(D)** Representative images of autophagesomes (red arrow) in Bel7402 control, sh1, and sh2 cells. Scale is 2 μm. Numbers of autophagosomes in three groups were quantified and analyzed. **(E)** Identification of the essential role of AMPK/mTOR signaling in autophagy induction in both Bel7402 shHMGB1 and HCCLM3 shHMGB1 HCC cells by AMPK siRNA transfection in (siAMPK). **(F)** Identification of HMGB1-mediated regulation of AMPK/mTOR signaling pathway in both Bel7402 and HCCLM3 cells by HMGB1 plasmid transfection (pEnter-HMGB1, 2 μg/ml). Data are means ± SEM from three independent experiments, * means p<0.05, ** means p<0.01, *** means p<0.001 by unpaired student T test.

To determine whether elevated HMGB1 was responsible for impaired autophagy, HMGB1 was knockdown by well-designed lentivirus shRNA in Bel7402 and HCCLM3 cell lines. The efficiency of HMGB1 knockdown was examined by immunoblot and Q-PCR analysis (**Figures 2A, B**). Interestingly, targeting HMGB1 could significantly prompt autophagic activity both in Bel7402 and HCCLM3 cell lines. To further demonstrate the effects of HMGB1 ablation in autophagy induction, experiments of immunofluorescence staining LC3B were conducted to visualize the distribution of LC3B puncta. As shown in **Figure 2C**, compared to cells with normal HMGB1, more LC3B puncta was detected in HMGB1 deficient cells. Transmission electron microscopy analysis confirmed that HMGB1 knockdown promoted the autophagosome formation (**Figure 2D**). These findings indicated that autophagic activity could be increased by targeting HMGB1.

Several signaling pathway such as PI3K/AKT/mTOR and AMPK/mTOR were reported to be involved in autophagy induction (34, 35). We observed that AMPK/mTOR signaling pathway was activated in HMGB1 deficient HCC cells and AMPK siRNA treatment efficiently abolished the autophagy upregulation, which indicated AMPK/mTOR signaling pathway was essential for HMGB1 deficiency-mediated autophagy induction (**Figure 2E**). Moreover, overexpressing HMGB1 in both Bel7402 and HCCLM3 cells resulted in decrease of autophagy level and inactivation of AMPK/mTOR signaling pathway (**Figure 2F**). Taking together, these findings indicated that HMGB1 repressed autophagic activity through modulate AMPK/mTOR signaling.

### Autophagy Is Responsible for HMGB1 Deficiency-Mediated HCC Inhibition

**Figure 3:**
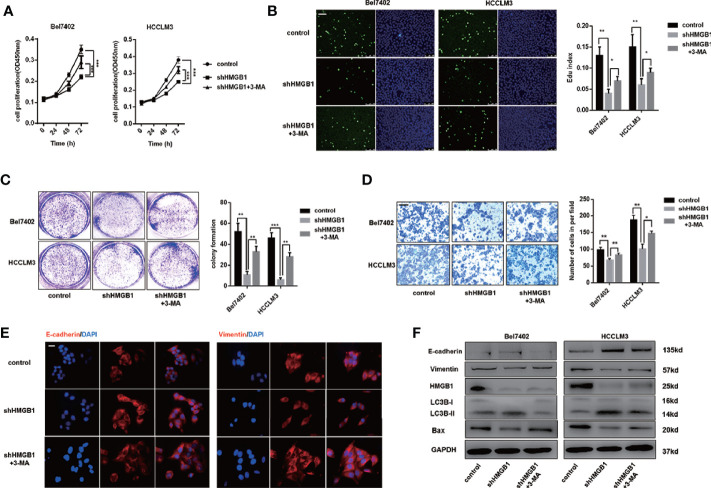
HMGB1 deficiency inhibits HCC development *via* autophagy induction. **(A)** Cell proliferation of HCC cells transfected as described, was determined by CCK-8 assays. 3-MA treatment (5mM) partly recovered damaged proliferation capacity of shHMGB1 cells. **(B)** Representative images of HCC cells stained by Edu assays. Numbers of Edu positive cells were counted and analyzed. 3-MA treatment (5 mM) partly recovered damaged Edu index of shHMGB1 cells. Scale is 400 μm. **(C)** Experiments of colony formation were performed in 12-well plates and results were analyzed. **(D)** Invasive capacity of HCC cells were determined by transwell experiments. Numbers of invaded cells were counted and analyzed. 3-MA treatment (5 mM) partly recovered impaired invasive capacity of shHMGB1 cells. Scale is 100 μm. **(E)** Representative images of IF staining E-cadherin and Vimentin in HCC cells with varied treatments, scale is 100 μm. **(F)** Immunoblot analysis of E-cadherin, Vimentin, BAX and autophagy markers was performed. 3-MA treatment (5 mM) partly recovered impaired EMT phenotype and BAX expression of shHMGB1 cells. Data are means ± SEM from three independent experiments, * means p<0.05, ** means p<0.01, *** means p<0.001 by unpaired student T test.

There is accumulating evidence that targeting HMGB1 suppresses tumor progression including proliferation, invasion and metastasis (29, 36). In **Figures S3A, B**, IHC staining analysis indicated that HMGB1 was positively associated with Ki67 and EMT phenotype (**Figures S3A, B**). To explore whether autophagy was involved in growth inhibition in HMGB1 deficient HCC cells, those cells were treated with 3-methyladenine (3-MA), an inhibitor repressing autophagesome formation. With the treatment of 3-MA, the inhibitory effect on proliferation observed in HMGB1 deficient cells was abrogated (**Figure 3A**), and impaired Edu index was partly rescued (**Figure 3B**). To further confirm these findings, experiments of colony formation were carried out. Consistently, 3-MA treatment dramatically rescued the damaged proliferative capacity of HMGB1 deficient cells (**Figure 3C**).

To gain more insights of the role of autophagy in HMGB1 deficient HCC cells, we conducted transwell experiments and demonstrated that 3-MA treatment recovered the invasive capacity(**Figure 3D**). Moreover, impaired EMT phenotype in HMGB1 deficient HCC cells were restored by inhibiting autophagy (**Figure 3E**). To confirm the anti-HCC effects of autophagy in HMGB1 knockdown HCC cells, immunoblot analysis was performed and results indicated that suppression of autophagy with 3-MA treatment could significantly recover the damaged expression of Bax and EMT related markers (**Figure 3F**). Besides, we observed similar results with the addition of specific autophagy inhibitor-chloroquine (CQ; **Figure S4**). Collectively, these findings demonstrated the critical role of autophagy in anti-HCC effects caused by HMGB1 inhibition.

### HMGB1 Interacts With HIPK2 and Promotes Its Protein Disability in HCC

**Figure 4:**
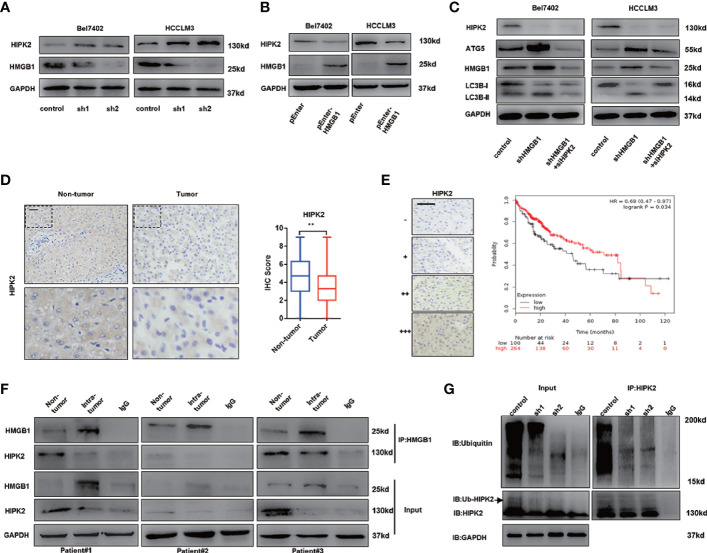
HMGB1 promotes the protein stability of HIPK2. **(A)** Protein levels of HIPK2 were determined in two HCC cell lines with a non-targeting control lentivirus-shRNA (control) or two lentivirus-shRNA targeting HMGB1 mRNA (sh1, sh2). **(B)** Protein levels of HIPK2 were determined in two HCC cell lines transfection with plasmid control (control) or plasmid HMGB1 (pEnter-HMGB1, 2μg/ml). **(C)** Identification of the essential role of HIPK2 in autophagy induction in both Bel7402 shHMGB1 and HCCLM3 shHMGB1 HCC cells by HIPK2 siRNA transfection in (siHIPK2). **(D)** Representative images of IHC staining HIPK2 in non-HCC and HCC tissue, scale is 100 μm. Quantification of HIPK2 levels according to IHC scores in non-tumor and tumor tissue. **(E)** Representative images of HIPK2 levels in HCC tissues, scale is 100 μm. Kaplan-Meier survival analysis of HCC patients based on HIPK2 levels by using public data (http://kmplot.com/analysis/index.php?p=service&cancer=liver_rnaseq). **(F)** Identification of the HMGB1-HIPK2 interaction in non-HCC and HCC tissue from three patients by co-immunoprecipitation. Endogenous HIPK2 was pulled down with anti-HMGB1, compared with IgG, and vice versa and detected by immunoblotting. **(G)** Immunoprecipitation of HIPK2 was performed with lysates of Bel7402 cells treated by a non-targeting control lentivirus-shRNA (control) or two lentivirus-shRNA targeting HMGB1 mRNA (sh1, sh2). Ubiquitination of precipitated HIPK2 was determined by Western blotting. Data are means ± SEM from three independent experiments, * means p<0.05, ** means p<0.01, *** means p<0.001 by unpaired student T test.

Impaired autophagy was demonstrated and associated with tumor progression in HCC (16–18). Our former data reveal that the tumor-promoting protein HMGB1 was involved in regulation of autophagy *via* AMPK/mTOR signaling and verified anti-HCC effects of HMGB1 inhibition accounted for autophagy induction. HIPK2 has been reported to be an important tumor suppressor and downregulated in varied malignances (37–39). Interestingly, we observed that targeting HMGB1 dramatically upregulated the protein expression of HIPK2 in HCC (**Figure 4A**). Moreover, the protein expression of HIPK2 was repressed in HMGB1 overexpressing HCC cells (**Figure 4B**), which indicated that there was a negative correlation between HMGB1 and HIPK2. To explore whether HIPK2 was involved in HMGB1 deficiency-mediated autophagy induction, HIPK2 siRNA was transfected in two shHMGB1 cell lines. With the silence of HIPK2, levels of autophagy were correspondingly downregulated, which suggested that targeting HMGB1 resulted in autophagy induction in a HIPK2-dependent way (**Figure 4C**).

To investigate the expression of HIPK2 in HCC, IHC staining HIPK2 was performed and IHC staining analysis showed HIPK2 expression was downregulated in HCC, compared to non-tumor tissues (**Figure 4D**). Meanwhile, HIPK2 expression was positively correlated with prognosis in HCC patients, which further confirmed the anti-HCC effects of HIPK2 (**Figure 4E**).

To further explore the relationship of HMGB1 and HIIPK2, Co-IP experiments were carried out. As shown in **Figure 4E**, HMGB1 expression was inversely correlated with HIPK2 and HMGB1 physically interacted with HIPK2 both in tumor or non-tumor tissues (**Figure 4F**).

Moreover, we observed that targeting HMGB1 inhibited the ubiquitination of HIPK2, which may promote HIPK2 protein stability (**Figure 4G**).

Our findings indicated that HMGB1 could interact with HIPK2 and targeting HMGB1 resulted in the de-ubiquitination of HIPK2. Moreover, HIPK2 was verified to be responsible for autophagy induction in HMGB1 deficient HCC cells.

### Autophagy Induction Results From HIPK2-Mediated Dysfunctional Glucose Metabolism

**Figure 5:**
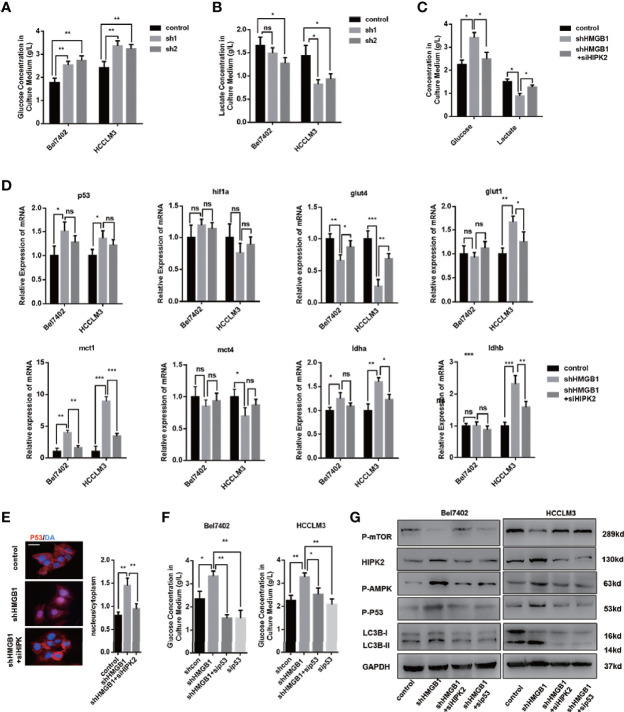
HIPK2 regulates autophagy levels in HMGB1 deficient cells *via* restricting glucose uptake. **(A)** Glucose concentration in cultural medium collected from two HCC cell lines with a non-targeting control lentivirus-shRNA (control) or two lentivirus-shRNA targeting HMGB1 mRNA (sh1, sh2). **(B)** Lactate concentration in cultural medium collected from cells as described. **(C)** Role of HIPK2 in impaired glucose uptake in shHMGB1 cells was determined by transfection with HIPK2 siRNA (siHIPK2). **(D)** Q-PCR experiments were performed to detect mRNA expressions of gene related to glucose metabolism in control, shHMGB1 and shHMGB1+siHIPK2 cells. **(E)** Representative images of IF staining p53 in control, shHMGB1 and shHMGB1+siHIPK2 cells. The distribution of p53 in cytoplasm or nucleus was quantified and analyzed. **(F)** Role of p53 in impaired glucose uptake in shHMGB1 cells was determined by transfection with p53 siRNA (sip53). Scale is 100 μm. **(G)** Protein levels of autophagy markers, AMPK/mTOR and p53 were determined by Western blotting. Data are means ± SEM from three independent experiments, NS means no significance. * means p<0.05, ** means p<0.01, *** means p<0.001 by unpaired student T test.

It has been elucidated that in the case of energy supplying by ATP, ubiquitination starts with the activation of ubiquitin molecules by ubiquitin-activating enzyme E1 activates ubiquitin molecules (40). An increase of the AMP/ATP ratio caused by ischemia activated AMPK, which was considered an essential mediator in cardiac ATP metabolism (41, 42). AMPK acts as an energy sensor and modulates cellular metabolism (43). When ATP production is damaged, AMPK is activated to stimulate ATP-generating pathways and restrict ATP-consuming pathways. Moreover, HMGB1 is essential for ATP production in both fibroblasts and tumor cells by regulating HSPB1 (44). Both AMPK/mTOR signaling and HIPK2 were confirmed to be essential for autophagy induction in HMGB1 deficient cells. To investigate the activation of AMPK, we assumed that HMGB1 deficiency leads to dysfunctional metabolism in a HIPK2-dependent way. Thus, we evaluated glucose and lactate levels in the culture medium to test our hypothesis. As shown in **Figures 5A, B**, HMGB1 ablated HCC cells exhibited impaired glucose uptake and lactate excretion. Moreover, treatment with HIPK2 siRNA dramatically recovered glucose uptake, indicating that targeting HMGB1-mediated HIPK2 upregulation was responsible for impaired glucose metabolism (**Figure 5C**).

Furthermore, we analyzed the expression levels of genes associated with glucose metabolism, and the results agreed with the impaired cellular glucose metabolism phenotype (**Figure 5D**). With the treatment of HIPK2 siRNA, impaired glucose metabolism was partly rescued (**Figures 5D**, **S5**). Besides, we observed an evident decline in the glucose transporter glut4 and a marked increase of p53 in HMGB1 knockdown cells (**Figure 5D**). It has been reported that p53 down-regulates glucose transporters GLUT4 gene expression, resulting in decreased glucose metabolism (45). Given the complex association between HMGB1 and p53, we then investigated the role of p53 and found that HMGB1 depletion triggered p53 translocation from the cytoplasm to the nucleus (**Figure 5E**). HIPK2 has been reported to promote p53 translocation by phosphorylation. With the treatment of HIPK2 siRNA, we obviously observed that p53 nuclear translocation was suppressed (**Figure 5E**). Moreover, after treatment with p53 siRNA, the dysfunctional glucose metabolism in shHMGB1 cells was restored, which indicated p53 was essential for HIPK2-mediated dysfunctional glucose metabolism in HMGB1 deficient cells (**Figure 5F**). Both two shHMGB1 HCC cells treated with HIPK2 siRNA or p53 siRNA, AMPK/mTOR signaling pathway and autophagy induction were inhibited (**Figure 5G**).

Collectively, these findings indicated HMGB1 deficient cells were characterized with impaired glucose uptake and HIPK2/p53 axis was essential for the dysfunctional glucose metabolism phenotype. Furthermore, HIPK2-mediated p53 nuclear translocation is responsible for activation of AMPK/mTOR signaling pathway.

### Autophagic Degradation of ZEB1 Is Responsible for HMGB1 Depletion-Mediated Inhibition of HCC

**Figure 6:**
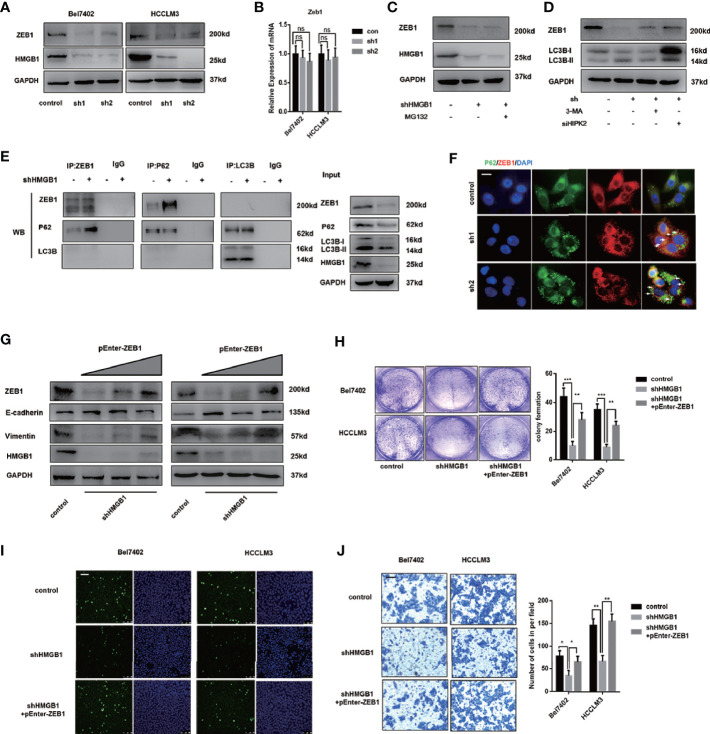
Autophagic degradation of ZEB1 is essential for HMGB1 deficiency-mediated HCC inhibition. **(A)** Protein levels of ZEB1 were determined in two HCC cell lines with a non-targeting control lentivirus-shRNA (control) or two lentivirus-shRNA targeting HMGB1 mRNA (sh1, sh2) by wetern blotting. **(B)** Q-PCR experiments were performed to detect ZEB1 mRNA expression in cells as described. **(C)** Bel7402 shHMGB1 cells were treated with MG132 (1μM). **(D)** Bel7402 shHMGB1 cells were treated with 3-MA (5mM) or HIPK2 siRNA respectively. **(E)** Identification of the ZEB1-p62 interaction in HCC cells by co-immunoprecipitation. Endogenous ZEB1 was pulled down with anti-p62 and endogenous p62 was pulled down with anti-ZEB1, compared with IgG, and vice versa and detected by immunoblotting. **(F)** Representative images of IF staining p62 and ZEB1 in HCC cells with a non-targeting control lentivirus-shRNA (control) or two lentivirus-shRNA targeting HMGB1 mRNA (sh1, sh2). Scale is 100 μm. **(G)** Protein levels of E-cadhein, Vimentin and ZEB1 were determined by wetern blotting. Plasmid ZEB1-cDNA was transfected in shHMGB1 cells in dose-dependent manner (pEnter-ZEB1, 0, 2, 4 μg/ml). **(H)** Cell proliferation of HCC cells transfected as described, was determined by colony formation experiments 12-well plates. **(I)** Representative images of HCC cells stained by Edu assays. Numbers of Edu positive cells were counted and analyzed. Scale is 400 μm. **(J)** Invasive capacity of HCC cells were determined by transwell experiments. Numbers of invaded cells were counted and analyzed. Scale is 100 μm. Data are means ± SEM from three independent experiments, NS means no significance. * means p<0.05, ** means p<0.01, *** means p<0.001 by unpaired student T test.

To further explore the mechanism on anti-HCC effects of HMGB1 inhibition, immunoblot analysis of EMT associated transcription factors was performed and results indicated that ZEB1 was the most downregulated in HMGB1 deficient HCC cells (**Figure 6A**). Interestingly, the mRNA expression of ZEB1 remained unchanged with the depletion of HMGB1, which indicated that the stability of ZEB1 protein was affected (**Figure 6B**). Moreover, following the treatment of MG132, a typical proteasome inhibitor, we found the protein expression of ZEB1 in HMGB1 deficient cell was not re-expressed, which suggested the protein instability of ZEB1 was not caused by the ubiquitin-proteasome system (**Figure 6C**). To determine whether autophagy was involved in the ZEB1 degradation, shHMGB1 HCC cells were treated with 3-MA, CQ or HIPK2 siRNA, respectively. As shown in **Figures 6D** and **S7**, autophagy induced by HMGB1 depletion accounted for ZEB1 decrease.

Through Co-IP experiments, we observed that p62 physically interacted with ZEB1 and the interaction was enhanced in HMGB1 deficient cells (**Figure 6E**). To further confirm this observation, immunofluorescence staining p62 and ZEB1 was carried out to visualize their distribution. Unexpectedly, targeting HMGB1 reinforced the formation of p62-ZEB1 complex (**Figure 6F**).

To verify the role of ZEB1 in HMGB1 depletion-mediated inhibition of HCC, we re-expressed ZEB1 in shHMGB1 cells and demonstrated that with the increase of ZEB1 expression, the impaired EMT phenotype was significantly rescued (**Figure 6G**). Furthermore, the capacities of proliferation and invasion in HMGB1 deficient cells were both recovered *via* ZEB1 re-expression (**Figures 6H–J**). Taken together, these results indicated that targeting HMGB1 lead to autophagy-induced selective degradation of ZEB1 by promoting the p62-ZEB1 complex formation and demonstrated that ZEB1 decrease was responsible for growth inhibition of HCC.

### Siah2 Is Responsible for HMGB1 Depletion-Induced ZEB1 Degradation

**Figure 7:**
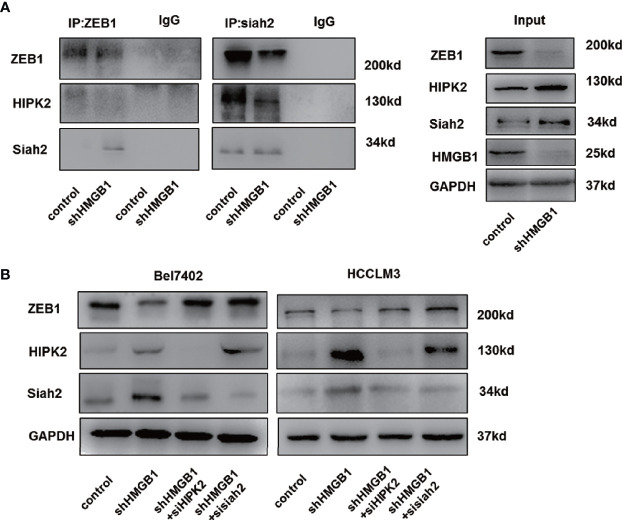
siah2 is essential for autophagic degradation of ZEB1 in HMGB1 deficient cells. **(A)** Identification of the ZEB1-siah2 interaction or HIPK2-siah2 interaction in HCC cells by co-immunoprecipitation. Endogenous ZEB1 or HIPK2 was pulled down with anti-siah2 and endogenous siah2 was pulled down with anti-ZEB1, compared with IgG, and vice versa and detected by immunoblotting. **(B)** Role of siah2 in ZEB1 decrease in shHMGB1 cells was determined by transfection with siah2 siRNA (sisiah2).

Siah2, an E3 ligase, has been reported to be essential for HIPK2 protein stability (46–48). Here, we found that siah2 was increased in HMGB1 deficient cells. Results from Co-IP experiments demonstrated the interaction of siah2 and HIPK2 was suppressed in shHMGB1 cells, which might explain why targeting HMGB1 inhibited the HIPK2 ubiquitination and upregulated HIPK2 protein expression (**Figure 7A**). Moreover, we observed that siah2 was directly combined to ZEB1 and siah2 was essential for HMGB1 inhibition-mediated ubiquitination of ZEB1 (**Figure 7A**). Following the treatment of siah2 siRNA, autophagic degradation of ZEB1 was inhibited in shHMGB1 cells (**Figure 7B**). Collectively, siah2 was essential for ZEB1 degradation caused by HMGB1 inhibition.

### HMGB1 Inhibitor Promotes Autophagic Decrease of ZEB1 and Rapamycin Enhanced the Anti-HCC Effects of HMGB1 Inhibitor

**Figure 8:**
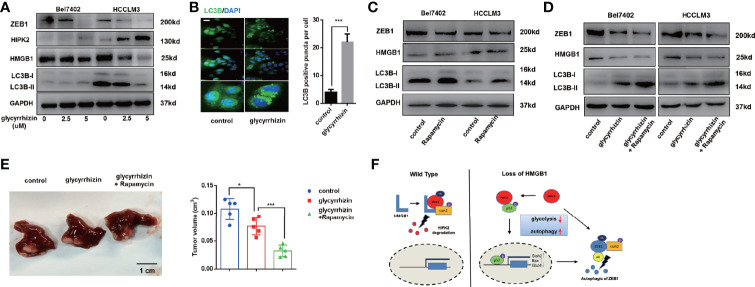
HMGB1 inhibitor exerts more efficient anti-HCC effects in combination with Rapamycin. **(A)** Related protein levels were determined in HCC cells treated with HMGB1 inhibitor glycyrrhizin (2.5 mM). **(B)** Representative images of IF staining LC3B in Bel7402 cells treated with or without glycyrrhizin (2.5 mM). Scale is 100 μm. **(C)** Related protein levels were determined in HCC cells treated with autophagy inducer Rapamycin (100 nM). **(D)** Rapamycin treatment enhanced the anti-HCC effects of glycyrrhizin. **(E)** Identification the inhibiting role of glycyrrhizin (50 mg/kg, i.p., twice every week for 4 weeks) with or without Rapamycin (10 mg/kg, i.p., twice every week for 4 weeks) *in vivo*. n=5. **(F)** Schematic depicting the role of HMGB1 in the regulation of HCC progression.

Glycyrrhizin, a HMGB1 inhibitor, has been demonstrated to inhibit HCC progression *via* disturbing hippo signaling pathway (29). Here, we observed glycyrrhizin treatment obviously repressed ZEB1 expression *via* HIPK2-mediated autophagy induction (**Figure 8A**). Analysis of immunofluorescence staining LC3B confirmed accumulating LC3B puncta in glycyrrhizin-treated HCC cells (**Figure 8B**). Though causing a significant upregulation of autophagy, the treatment of Rapamycin, an autophagy inducer, did not promote the decrease of ZEB1, which indicated that autophagic degradation of ZEB1 occurred in special conditions like HMGB1 inhibition (**Figure 8C**). However, Rapamycin treatment could reinforce glycyrrhizin-mediated decrease of ZEB1 *in vitro* and growth inhibition of HCC *in vivo* (**Figures 8D, E**). Taken together, these data revealed HMGB1 inhibitor glycyrrhizin suppressed HCC progression by decreasing ZEB1 and the combination of Rapamycin and glycyrrhizin was a more effective treatment than glycyrrhizin only.

## Discussion

In this study, we gained several insights into the mechanism of HCC progression: (1) In HCC, HMGB1 expression is negatively correlated with autophagy levels and HMGB1 regulates autophagy *via* AMPK/mTOR signaling. Thus, HMGB1 overexpression is responsible for autophagy decrease in HCC; (2) Targeting HMGB1 results in autophagy induction through promoting HIPK2 protein stability and HIPK2/p53 axis accounts for suppression of glucose uptake in HMGB1 deficient cells; (3) Targeting HMGB1 causes ZEB1 decrease through promoting p62-ZEB1 complex formation and ZEB1 decrease is responsible for anti-HCC effects of HMGB1 inhibition; (4) Siah2 interacts to ZEB1 and is essential for ZEB1 decrease in HMGB1 deficient cells; (5) HMGB1 inhibitor glycyrrhizin inhibits HCC progression *via* decreasing ZEB1 and rapamycin treatment could reinforce glycyrrhizin-mediated HCC growth inhibition.

Autophagy is an important cellular process, which has complicated effects during tumorigenesis (12, 49). Increasing evidence suggests that the role of autophagy in tumors is controversial because autophagy is involved in both tumor promotion and suppression. Accumulating data demonstrate that impaired autophagy has a critical role in many aspects of tumor progression, including HCC progression. The inhibition of autophagy results in the accumulation of the tumor-promoting protein p62, thereby leading to tumors’ initiation. Interestingly, we observed down-regulation of HMGB1 increased p62, which is usually considered an autophagy degradation substrate at the transcriptional level (**Figure 2B**). At protein levels, the upregulation of autophagy marker proteins (ATG5, Beclin-1 and LC3B) and a corresponding reduction of autophagy substrate p62 indicate that autophagy level is increased (34, 50). It has been elucidated that p62 possesses multiple structural domains and interacts with LC3 for autophagosome biosynthesis, allowing p62 to play a critical role in autophagy (51). Besides, the localization of p62 to the autophagosome formation site requires the PB1 domain-dependent self-oligomerization, which indicates p62-LC3 binding may not be sufficient for autophagy (52). Also, it is reported that proteasome inhibition (another competitive protein degradation mechanism of autophagy degradation) and starvation also induced p62 synthesis, while cleavage of sequestosome 1/p62 resulted in disrupted selective autophagy (53, 54). These findings indicated that p62 might not only act as a substrate of autophagic degradation, and the role of p62 in HMGB1-related autophagy in HCC also needs further research. However, tumor cells also sustain proliferation by inducing autophagy to facilitate autophagy-mediated survival, drug resistance, and invasion. Our study revealed that impaired autophagy was associated with HCC prognosis and HMGB1 overexpression. Moreover, we verified that HMGB1 modulated autophagy levels through AMPK/mTOR signaling pathway. Our further study suggested that autophagy induction was essential for HMGB1 inhibition-mediated anti-HCC effects.

HMGB1 has been demonstrated a tumor-promoting protein and involved in many aspects of tumor progression including proliferation, invasion, metastasis and drug resistance (28). Previous studies have shown that HMGB1 overexpression in HCC facilitates HCC invasion by regulating caspase-1 and miR-21 (36, 55). Moreover, under starvation conditions, HMGB1 is released from the nucleus and binds to beclin-1 (15). The formation of the HMGB1-beclin-1 complex is responsible for the induction of autophagy to maintain cell survival in stressed situations. In this study, we showed that targeting HMGB1 inhibited HCC progression *via* stabilizing HIPK2 protein. We observed that HIPK2 expression was downregulated in HCC and inversely associated with HMGB1. HMGB1 physically interacted with HIPK2 and HMGB1 deficiency inhibited HIPK2 ubiquitination. Moreover, HIPK2 was determined to be essential for growth inhibition in HMGB1 deficient HCC cells.

Previous study identifies HMGB1 as an essential mediator of mitochondrial quality control by increasing HSPB1 (44). In the absence of HMGB1, mouse embryonic fibroblasts exhibit mitochondrial fragmentation, dysfunctional mitochondrial respiration, and low ATP production, thereby suggesting that HMGB1 is essential for cellular metabolism. Compared with control cells, we found that HMGB1 deficient HCC cells exhibited defective glucose uptake, and the expression levels of glycolysis-associated genes were decreased. Low ATP production is a critical signal that activates the AMPK signaling pathway. Meanwhile, we demonstrated HIPK2/p53 axis was responsible for impaired glucose uptake in HMGB1 deficient cell, as a result of which, autophagy mediated by AMPK/mTOR signaling was triggered.

Selective degradation is an important function of autophagy. Previous studies have shown that damaged autophagic degradation of miR-224 facilitates HCC proliferation and invasion *via* TGF-β/smad signaling pathway (17). We demonstrated the direct interaction between p62 and ZEB1 and observed the enhanced formation of p62-ZEB1 complex in HMGB1 deficient cells. Furthermore, we confirmed that ZEB1 decrease was essential for anti-HCC effects by HMGB1 inhibition.

HIPK2 has been shown to be targeted and ubquitinated by siah2 (46, 48). In our study, we confirmed that HIPK2 and ZEB1 were both interacted with siah2. In the absence of siah2, HMGB1 inhibition-mediated decrease of ZEB1 was abolished. Siah2 has been reported to be transcriptionally activated by p53 and the interaction with HIPK2 could result in phosphorylation of siah2, which reinforces the E3 ligase. These evidences could provide some explanations for the upregulation of siah2 and siah2-mediated ubiquitination of ZEB1 in HMGB1 deficient HCC cells.

We also explored the effects on HCC progression by administration of HMGB1 inhibitor glycyrrhizin. Glycyrrhizin effectively inhibited HMGB1 expression and resulted in ZEB1 decrease. Rapamycin has been demonstrated as an autophagy inducer and tumor chemotherapy drug. Several studies indicate rapamycin represses varied tumor progression *via* autophagy. Though rapamycin failed to suppress ZEB1, it could significantly enhance HCC inhibition caused by glycyrrhizin.

In summary, our results demonstrate important roles of impaired autophagy mediated by HMGB1 in HCC progression *via* AMPK/mTOR signaling pathway. Targeting HMGB1 could result in autophagy induction by HIPK2/p53 axis. Furthermore, autophagic degradation of ZEB1 is determined to be responsible for HCC repression caused by targeting HMGB1. Lastly, we confirm the inhibitory effects of glycyrrhizin on HCC progression and indicate the combination therapy of glycyrrhizin and rapamycin gains more anti-HCC effects. These findings support the role of HMGB1/HIPK2/autophagy/ZEB1 axis in HCC development, and this novel mechanism may provide new therapeutic targets for HCC treatment.

## Data Availability Statement

The original contributions presented in the study are included in the article/**Supplementary Material**. Further inquiries can be directed to the corresponding authors.

## Ethics Statement

The present study was approved by the Ethics Committee of Nanjing Drum Tower Hospital (Nanjing, China) and written informed consent was provided by all patients. In vivo experiments were approved by the Institutional Animal Care and Use Committee of Nanjing University, China, based on the NIH Guide for the Care and Use of Laboratory Animals.

## Author Contributions

WZ, JL and YHZ performed experiments and analyzed data and wrote the manuscript. JL and WZ contributed in animal experiments and manuscript writing. HZR and XLS designed the project and coordinated the execution of the experimental plan. ZYZ, HYL, YZL, AYH and JCZ performed detailed methods and data validation. XLS obtained funding, and directed the study. All authors contributed to the article and approved the submitted version.

## Funding

The project was funded by grants from the National Natural Science Foundation of China (81872359, 81670566), Jiangsu Province’s Key Provincial Talents Program (ZDRCA2016066), the Nanjing Medical Science and Technique Development Foundation (QRX17129), the Nanjing health science and technology development project for Distinguished Young Scholars (JQX19002), the Nanjing Science and technology project (201911039), Innovation and entrepreneurship education incubation project of Nanjing University.

## Conflict of Interest

The authors declare that the research was conducted in the absence of any commercial or financial relationships that could be construed as a potential conflict of interest.

## Abbreviations

HMGB1, high-mobility group box 1; EMT, epithelial-mesenchymal transition; AMPK, Adenosine 5’-monophosphate (AMP)-activated protein kinase; mTOR, mammalian target of rapamycin; Rapa, rapamycin; 3-MA, 3-Methyladenine; CQ, chloroquine; PVTT, portal vein tumor thrombosis.
