# Supplementary material for: Inhibition of HMGB1 Suppresses Hepatocellular Carcinoma Progression via HIPK2-Mediated Autophagic Degradation of ZEB1
Source: Front Oncol. 2021 Mar 4;11:599124. doi: 10.3389/fonc.2021.599124 (PMC7969871; doi:10.3389/fonc.2021.599124)
Supplement: Supplementary file 1 [file DataSheet_1.docx]

**SUPPLEMENTARY MATERIALS**

| **Supplementary Table S1: The associations of HMGB1 expression with clinicopathological characteristics in HCC patients** | | | |
| --- | --- | --- | --- |
| Feature | Low expression  of HMGB1(n=31) | Hgh expression  of HMGB1(n=45) | P-value |
| Gender |  |  |  |
| Male | 22 | 37 | 0.27^a^ |
| Female | 9 | 8 |  |
| Age(years) |  |  |  |
| >=50 | 26 | 31 | 0.14 |
| <50 | 5 | 14 |  |
| HBsAg |  |  |  |
| Negative | 16 | 10 | **0.01^a^** |
| Positive | 15 | 35 |  |
| AFP(ng/ml) |  |  |  |
| >=20 | 20 | 26 | 0.55 |
| <20 | 11 | 19 |  |
| Cirrhosis |  |  |  |
| No | 21 | 23 | 0.15 |
| Yes | 10 | 22 |  |
| Tumor size(cm) | |  |  |
| >=5 | 19 | 23 | 0.38 |
| <5 | 12 | 22 |  |
| Tumor number |  |  |  |
| Single | 14 | 20 | 0.95 |
| Multiple | 17 | 25 |  |
| PVTT |  |  |  |
| No | 27 | 28 | **0.03^a^** |
| Yes | 4 | 16 |  |
| TNM stage |  |  |  |
| I+II | 23 | 22 | **0.03** |
| III+IV | 8 | 23 |  |
| Recurrence |  |  |  |
| No | 29 | 37 | 0.15 |
| Yes | 2 | 8 |  |
| Abbreviations: AFP, α -fetoprotein; HBsAg, hepatitis B surface antigen; HCC, hepatocellular carcinoma; HMGB1, High-mobility group box 1; PVTT, portal vein tumor thrombosis; ^a^Fisher's exact tests and Pearson's χ ^2^ tests for all the other analysis. | | | |

**Supplemental Table S2: qPCR primer sequences**

| Gene | Forward ­­ | Reverse |
| --- | --- | --- |
| 18s | CGGCG ACGACCCATTCGAAC | GAATCGAACC CTGAT TCCCC GTC |
| Hmgb1 | TATGGCAAAAGCGGACAAGG | CTTCGCAACATCACCAATGGA |
| Lc3II | AGCAGCATCCAACCAAAATC | CTGTGTCCGTTCACCAACAG |
| P62 | ATCGGAGGATCCGAGTGT | TGGCTGTGAGCTGCTCTT |
| Beclin1 | CAGGAGAGACCCAGGAGGAA | GCTGTTGGCACTTTCTGTGG |
| Zeb1 | CTACAACAACAAGACACTGCTGT | TGTTCTTTCAGAGAGGTAAAGCG |
| Hif1a | ATCCATGTGACCATGAGGAAATG | TCGGCTAGTTAGGGTACACTTC |
| P53 | CAGCACATGACGGAGGTTGT | TCATCCAAATACTCCACACGC |
| Siah2 | TCTTCGAGTGTCCGGTCTG | CGGCATTGGTTACACACCAG |
| Glut1 | GGCCAAGAGTGTGCTAAAGAA | ACAGCGTTGATGCCAGACAG |
| Glut4 | TGGGCGGCATGATTTCCTC | GCCAGGACATTGTTGACCAG |
| Mct1 | GGTGGAGGTCCTATCAGCAGT | CAGAAAGAAGCTGCAATCAAGC |
| Mct4 | AGGTATCCTTGAGACGGTCAG | CAAGCAGGTTAGTGATGCCG |
| ldha | TTGACCTACGTGGCTTGGAAG | GGTAACGGAATCGGGCTGAAT |
| ldhb | TCTGTGACCGCCAATTCTAAGA | GCACCAGATTGAGCCGACTC |
|  |  |  |
|  |  |  |
|  |  |  |
|  |  |  |
|  |  |  |
|  |  |  |
|  |  |  |
|  |  |  |

**Supplemental Figures and Figure legends**


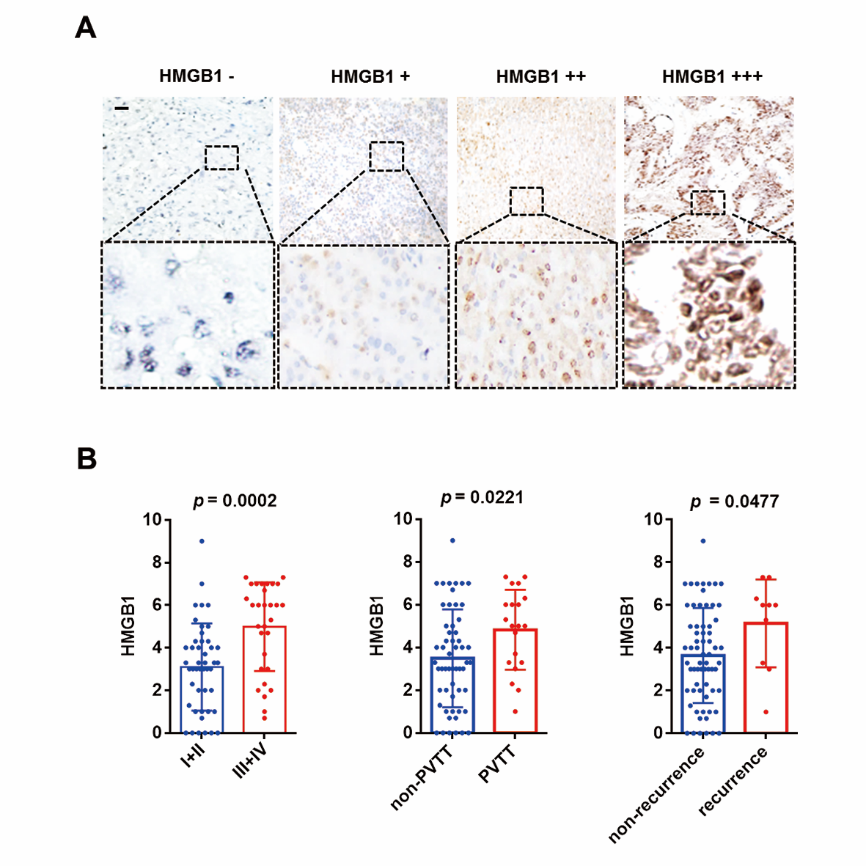


Fig.S1 Expression of HMGB1 is correlated with poor prognosis of HCC patients.

(A) Representative images of IHC staining HMGB1 in HCC samples, scale is100 μm. +,<25%; ++, <50%; +++, <75%; ++++, >75%. (B) Correlation of HMGB1 and clinical signatures, including TNM stage, PVTT and tumor recurrence.


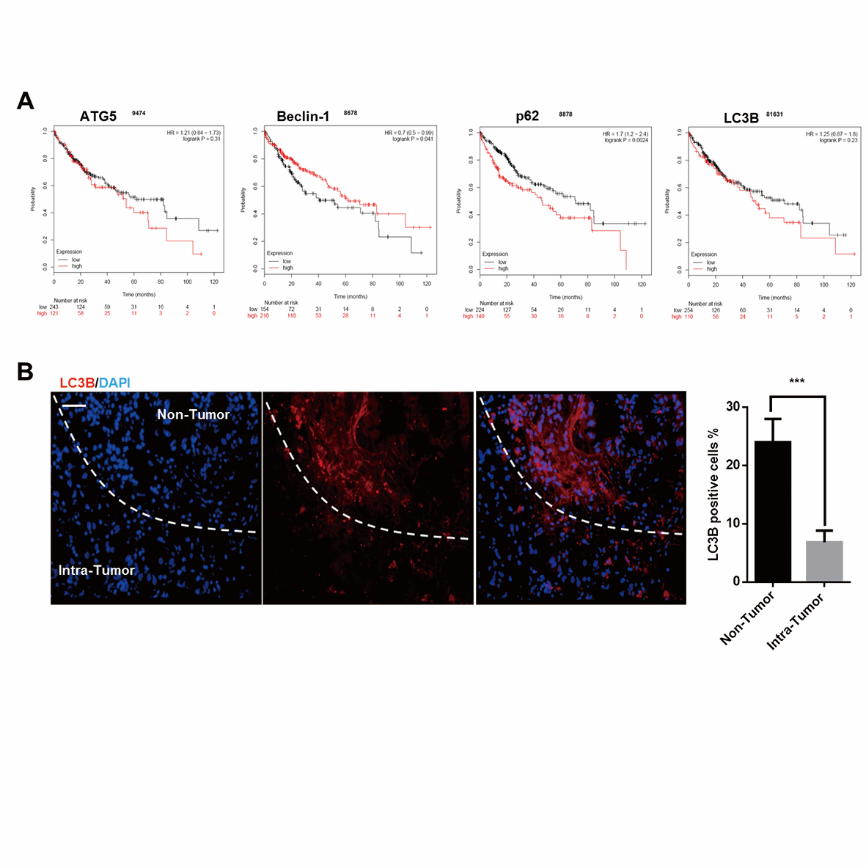


Fig.S2 Autophagy dysfunction is associated with poor prognosis of HCC patients

(A) Kaplan-Meier survival analysis of HCC patients based on several autophagy markers using public data (<http://kmplot.com/analysis/index.php?p=service&cancer=liver_rnaseq>). (B) Representative images of IF staining LC3B in HCC specimens. Numbers of LC3B positive cells in non-HCC or HCC tissues were counted and analyzed.


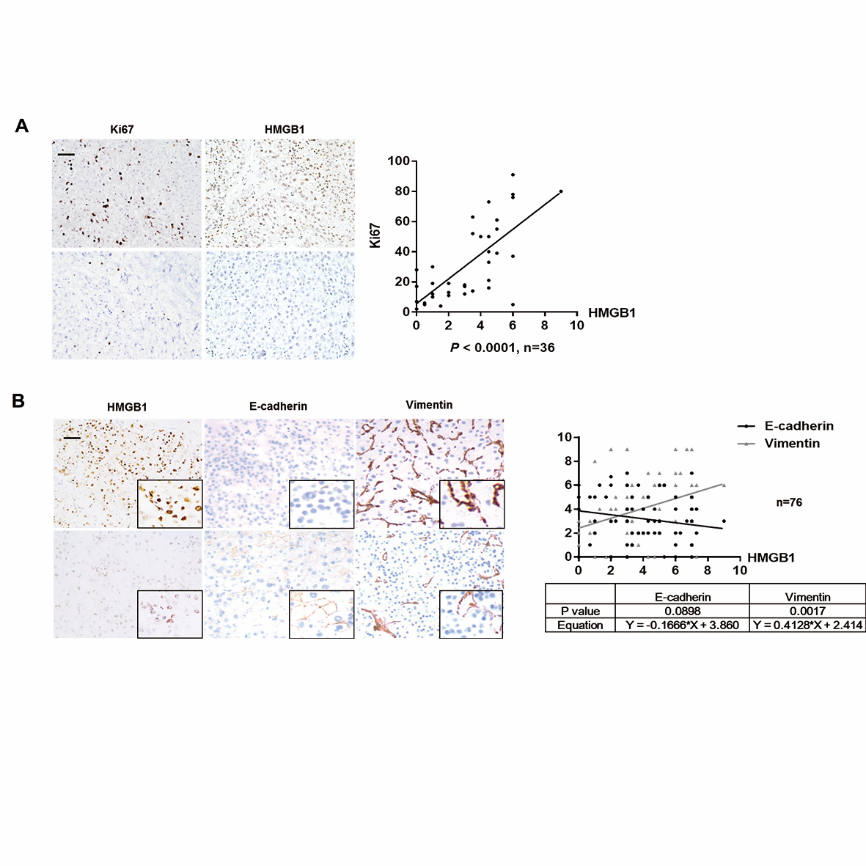


Fig.S3 HMGB1 expression is related to HCC proliferation and EMT phenotype.

(A) Representative images of IHC staining HMGB1 and Ki67 in HCC specimens. Analysis of IHC scores of HMGB1 and Ki67 was performed. (B) Representative images of IHC staining HMGB1, E-cadherin and Vimentin in HCC specimens. Analysis of IHC scores of HMGB1, E-cadherin and Vimentin was performed.


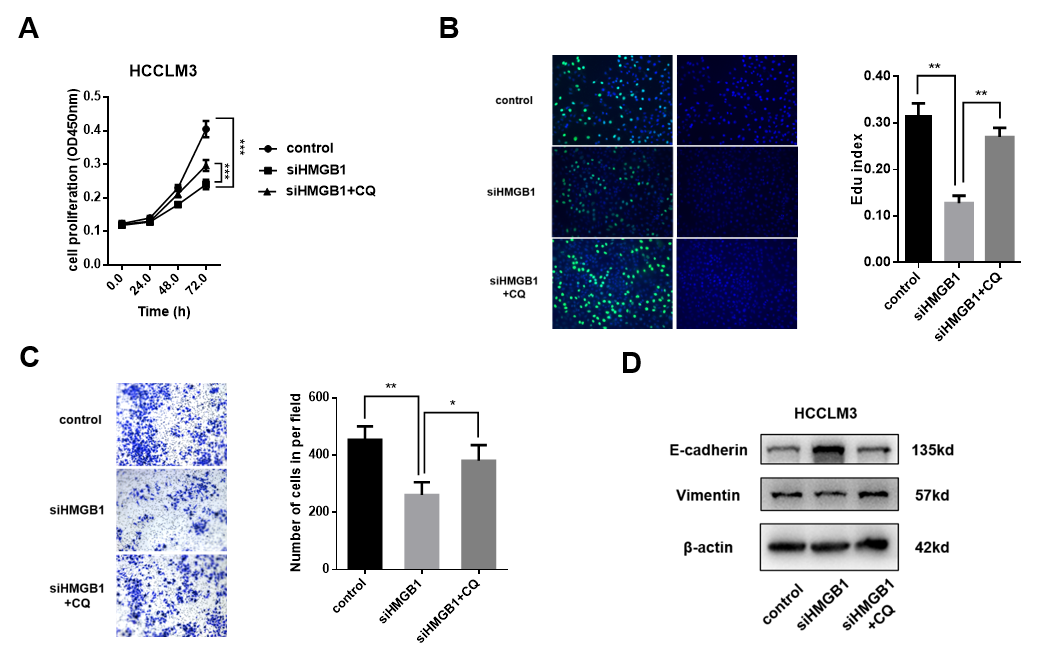


Fig.S4 The inhibition of autophagy diminishes HMGB1-knockdown reduced HCCLM3 proliferation and invasion.

(A) Cell proliferation of HCCLM3 cells transfected as described was determined by CCK-8 assays. CQ treatment (5mM) partly recovered the damaged proliferation capacity of siHMGB1 cells. (B) Representative images of HCCLM3 cells stained by Edu assays. Numbers of Edu positive cells were counted and analyzed. CQ treatment (5mM) partly recovered the damaged Edu index of siHMGB1 cells. The scale is 400μm. (C) Transwell experiments determined the invasive capacity of HCC cells. Numbers of invaded cells were counted and analyzed. CQ treatment (5mM) partly recovered the impaired invasive ability of siHMGB1 cells. The scale is 100μm. (D) Immunoblot analysis of E-cadherin and Vimentin was performed. CQ treatment (5mM) partly recovered impaired EMT phenotype of siHMGB1 cells. CQ, chloroquine. Data are means ± SEM from 3 independent experiments, * means p<0.05, ** means p<0.01, *** means p<0.001 by unpaired student T-test.


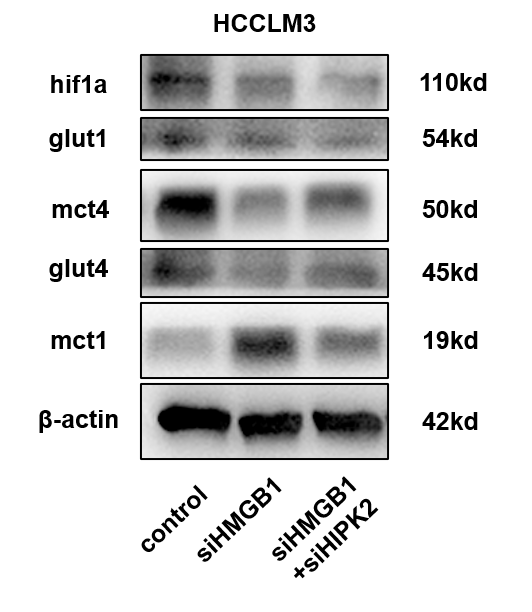


Fig.S5 HIPK2 regulates glucose uptake related proteins in HMGB1 deficient HCCLM3 cells.

Immunoblot analysis of representative glucose uptake related proteins in HMGB1 deficient HCCLM3 cells, with or without siHIPK2 transfection.


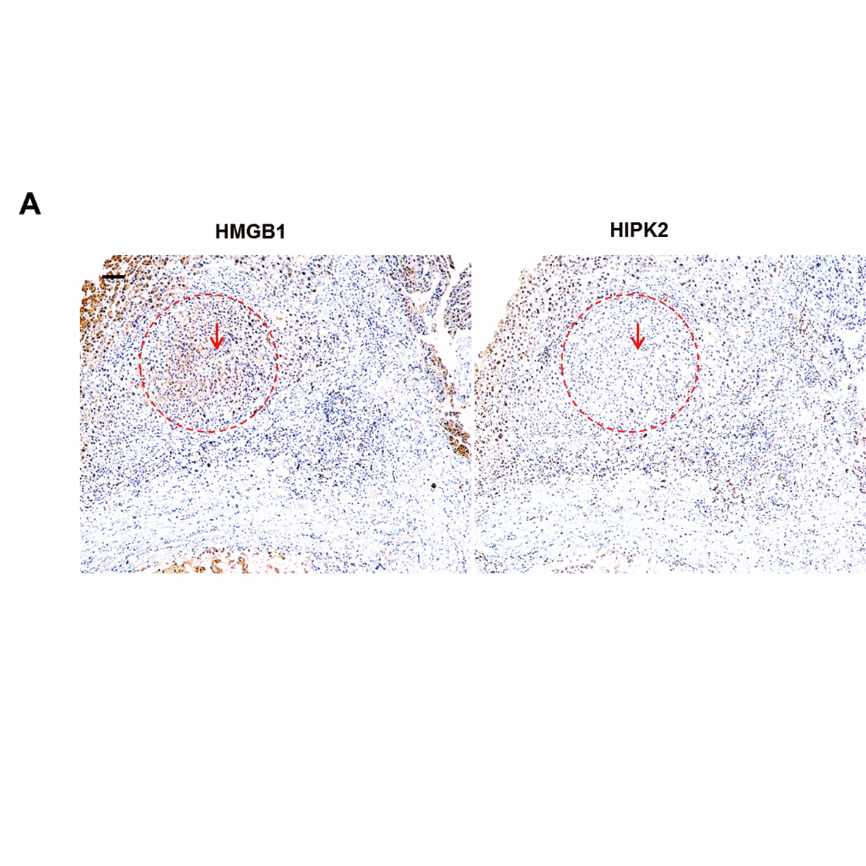


Fig.S6 HMGB1 expression is inversely correlated with HIPK2

(A) Representative images of IHC staining HMGB1 and HIPK2 in HCC specimens. A red arrow marked the same place in HCC tissues.


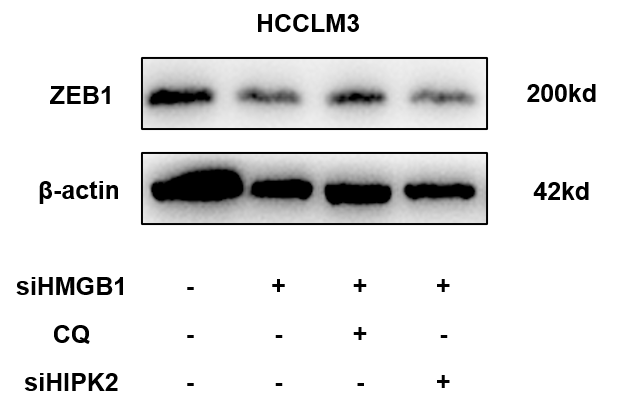


Fig.S7 The inhibition of autophagy reverses HMGB1-knockdown reduced ZEB1 in HCCLM3 cells.

Immunoblot analysis of ZEB1 protein level in HCCLM3 cells transfected with siHMGB1, with or without CQ (5mM) addition. CQ, chloroquine.
